# Letter to Editor: Postbiotics as a novel intervention for obesity management and improving metabolic parameters: a systematic review and meta-analysis of animal studies

**DOI:** 10.1186/s12967-025-07127-5

**Published:** 2025-10-24

**Authors:** Mahdieh Khodarahmi

**Affiliations:** Nutrition and Food Security Research Center, Isfahan University of Medical Sciences, Isfahan, Iran

Dear Editor,

We read with great interest the recent systematic review and meta-analysis by Nazarinejad et al., [1] which addressed the potential of postbiotics as a novel intervention for obesity management and improvement of metabolic parameters in animal studies. The topic is of considerable importance given the rising prevalence of obesity worldwide and the urgent need for effective, evidence-based therapeutic strategies.

While the analysis contributes valuable insights to the field, we noted that the article does not provide the reference list of included studies. This omission raises concerns regarding transparency, reproducibility, and the ability of readers to critically appraise the validity of the conclusions. In systematic reviews and meta-analyses, explicit reporting of included studies is fundamental to ensure that the evidence synthesis is both verifiable and replicable.

According to the PRISMA (Preferred Reporting Items for Systematic Reviews and Meta-Analyses) 2020 guidelines, systematic reviews should cite all included studies in the manuscript text, particularly in the Results section, when describing study characteristics and findings [2]. Furthermore, PRISMA recommends that authors present a list of included and excluded studies, ideally as supplementary material, to facilitate accountability and enable readers to independently assess the comprehensiveness and rigor of the review process [2]. Without this information, the scientific community cannot fully evaluate the quality of the evidence base, nor can future researchers efficiently build upon the work.

We respectfully suggest that the authors, or the journal, consider making the reference list of included studies available, for example as an online supplementary file. This would enhance the transparency, reproducibility, and overall impact of the meta-analysis, while aligning the article with internationally recognized reporting standards.

We commend the authors for addressing an emerging and promising area of research, and we believe that the provision of the included references will further strengthen the utility and credibility of their important contribution.

Sincerely,

Mahdieh Khodarahmi.
